# The Mendelian colorectal cancer syndromes

**DOI:** 10.1177/0004563215597944

**Published:** 2015-11

**Authors:** Ian Tomlinson

**Affiliations:** Molecular and Population Genetics Laboratory and Oxford NIHR Biomedical Research Centre, Wellcome Trust Centre for Human Genetics, University of Oxford, Oxford, UK

**Keywords:** Genetics, cancer, gastrointestinal disorders

## Abstract

A small minority of colorectal cancers (CRCs) (≤5%) are caused by a single, inherited faulty gene. These diseases, the Mendelian colorectal cancer (CRC) syndromes, have been central to our understanding of colorectal carcinogenesis in general. Most of the approximately 13 high-penetrance genes that predispose to CRC primarily predispose to colorectal polyps, and each gene is associated with a specific type of polyp, whether conventional adenomas (*APC*, *MUTYH*, *POLE*, *POLD1*, *NTHL1*), juvenile polyps (*SMAD4*, *BMPR1A*), Peutz-Jeghers hamartomas (*LKB1/STK11*) and mixed polyps of serrated and juvenile types (*GREM1*). Lynch syndrome (*MSH2*, *MLH1*, *MSH6*, *PMS2*), by contrast, is associated primarily with cancer risk. Major functional pathways are consistently inactivated in the Mendelian CRC syndromes: certain types of DNA repair (proofreading of DNA replication errors, mismatch repair and base excision repair) and signalling (bone morphogenetic protein (BMP), Wnt signalling and mTOR). The inheritance of the CRC syndromes also varies: most are dominant but some of the DNA repair deficiencies are recessive. Some of the Mendelian CRC genes are especially important because they play a role through somatic inactivation in sporadic CRC (*APC*, *MLH1*, *SMAD4*, *POLE*). Additional Mendelian CRC genes may remain to be discovered and searches for these genes are ongoing, especially in patients with multiple adenomas and hyperplastic polyps.

Most cases of colorectal cancer (CRC) arise as a result of somatic mutations in a stem-like cell somewhere in the epithelium of the large bowel. Most of these mutations seem to be spontaneous changes, consistent with the notion that cancer is mostly down to bad luck. However, we know that certain environments and common genetic polymorphisms can modify this risk, and up to one-third of the variation in CRC risk may have an inherited basis. In a small minority of CRC patients, moreover, there is a strong genetic predisposition caused by a single faulty gene.^1–5^ These diseases, the Mendelian CRC syndromes, have been important in furthering our understanding of colorectal carcinogenesis, and this work, in turn, has improved the management of these patients and their families. However, as evidenced by the case of Stephen Sutton, who had Lynch syndrome (see below), there is still sometimes an under-appreciation of the importance of inherited cancer risk and of the fact that some unfortunate carriers of Mendelian CRC genes can develop cancer as early as their second and third decades of life (http://www.bowelcanceruk.org.uk/never-too-young/never-too-young-reports/lynch-syndrome-testing-and-bowel-cancer/).

The complete wealth of data on the Mendelian CRC syndromes (Figure 1) cannot be reviewed here. Instead, I shall emphasize some of the features of this group of diseases that I find the most remarkable. The first thing of note is that there are several genetic routes to CRC. There are at least 13 known high-penetrance genes that predispose to CRC when mutated in the germ line. Most of these predispose primarily to colorectal polyps, and each gene is associated with a specific type of polyp, whether conventional adenomas (*APC*, *MUTYH*, *POLE*, *POLD1*, *NTHL1*), juvenile polyps (*SMAD4*, *BMPR1A*), Peutz-Jeghers hamartomas (*LKB1/STK11*) and mixed polyps of serrated and juvenile types (*GREM1*). It is not known whether the increased CRC risk in these conditions results from random progression of the polyps to cancer as they acquire random somatic mutations, although this is a plausible model. Lynch syndrome, by contrast, is not usually associated with an excess of polyps, but specifically with CRC risk. However, in some very rare individuals with congenital mismatch repair deficiency (CMMRD), who carry mutations in both copies of a Lynch syndrome gene (*MSH2*, *MLH1*, *MSH6*, *PMS2*), there is a predisposition to conventional adenomas.

**Figure 1.**
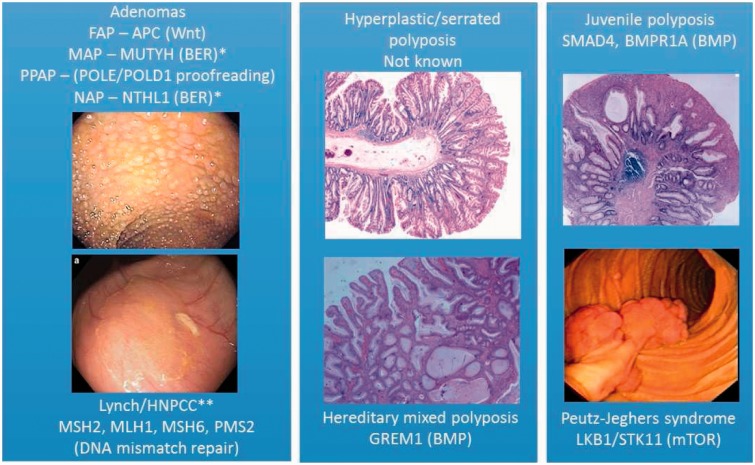
Mendelian colorectal cancer syndromes, showing the genes mutated in the germline of affected individuals (and the pathway in which they act) and the colorectal lesion to which there is a primary predisposition. Note that there is a greatly increased risk of CRC in all syndromes, and most have increased risks of benign and malignant extracolonic tumours. Only in Lynch syndrome is the primary predisposition to carcinomas, usually with few polyps. In the other syndromes, the polyp is the principal lesion, ranging from classical adenomas to pathognomonic lesions such as juvenile polyps and Peutz-Jeghers polyps. Note: *recessive; **also mutated in the recessive condition Congenital Mismatch Repair Deficiency, in which colorectal tumour predisposition is principally to adenomas.

Major functional pathways are consistently disrupted in Mendelian CRC syndromes. The first is repair of small mutations (base substitutions and small insertions/deletions). Polymerase proofreading-associated polyposis (PPAP) patients (Figure 1) have defects in proofreading of errors during DNA replication, Lynch syndrome results from defects in DNA mismatch repair, and *MUTYH*-associated polyposis (MAP) and *NTHL1*-associated polyposis (NAP) patients have reduced base excision repair. The tumours in these patients show a somatic mutation spectrum concordant with the types of mutation that are normally repaired by the pathway concerned. Why these forms of DNA repair and not, say, double-strand break repair, are associated with CRC risk remains unclear. Other CRC genes act in the bone morphogenetic protein (BMP) signalling pathway which is involved in controlling the differentiation of cells as they pass up the colorectal crypt from the stem cell zone. Juvenile polyposis and hereditary mixed polyposis have defective BMP signalling. Other pathways defective in CRC syndromes include Wnt signalling (familial adenomatous polyposis, *APC*) and mTOR (Peutz-Jeghers syndrome, *LKB1*). Why defects in such diverse pathways can lead to very similar phenotypes is unknown.

There is also variety in the inheritance of the CRC syndromes. Most are dominant, involving tumour suppressor genes with cell autonomous effects once the second copy has been randomly mutated. Of the DNA repair deficiencies, Lynch syndrome follows this model, but MAP, NAP and CMMRD are recessive – we do not know why this is the case. HMPS is also unusual in that *GREM1* acts as an oncogene and is a non-cell autonomous, secreted protein. There is ongoing controversy as to whether *SMAD4*, *BMPR1A* and *LKB1* act as tumour suppressors or are haploinsufficient.

Some of the Mendelian CRC genes are especially important because they play a role through somatic inactivation in sporadic CRC. *APC* is mutated in almost all CRCs, *MLH1* (but not the other Lynch genes) is silenced in ∼15% of CRCs, *SMAD4* is mutated in ∼10% of CRCs and *POLE* (but oddly not *POLD1*) is mutated in 2–3% of CRCs. Sometimes (*MLH1*, *POLE*) these changes are associated with good prognosis.

Additional Mendelian CRC genes may remain to be discovered. Searches for these genes in patients with multiple adenomas and hyperplastic/serrated polyposis syndrome^6^ are continuing.
